# Mechanisms of endometrial aging: lessons from natural conceptions and assisted reproductive technology cycles

**DOI:** 10.3389/fphys.2024.1332946

**Published:** 2024-02-28

**Authors:** Anat Chemerinski, Jessica Garcia de Paredes, Kristin Blackledge, Nataki C. Douglas, Sara S. Morelli

**Affiliations:** Department of Obstetrics, Gynecology and Reproductive Health, Rutgers New Jersey Medical School, Newark, NJ, United States

**Keywords:** endometrium, aging, senescence, pregnancy, assisted reproductive technology, endometrial microbiome, endometrial receptivity

## Abstract

Until recently, the study of age-related decline in fertility has focused primarily on the ovary; depletion of the finite pool of oocytes and increases in meiotic errors leading to oocyte aneuploidy are well-established mechanisms by which fertility declines with advancing age. Comparatively little is known about the impact of age on endometrial function. The endometrium is a complex tissue comprised of many cell types, including epithelial, stromal, vascular, immune and stem cells. The capacity of this tissue for rapid, cyclic regeneration is unique to this tissue, undergoing repeated cycles of growth and shedding (in the absence of an embryo) in response to ovarian hormones. Furthermore, the endometrium has been shown to be capable of supporting pregnancies beyond the established boundaries of the reproductive lifespan. Despite its longevity, molecular studies have established age-related changes in individual cell populations within the endometrium. Human clinical studies have attempted to isolate the effect of aging on the endometrium by analyzing pregnancies conceived with euploid, high quality embryos. In this review, we explore the existing literature on endometrial aging and its impact on pregnancy outcomes. We begin with an overview of the principles of endometrial physiology and function. We then explore the mechanisms behind endometrial aging in its individual cellular compartments. Finally, we highlight lessons about endometrial aging gleaned from rodent and human clinical studies and propose opportunities for future study to better understand the contribution of the endometrium to age-related decline in fertility.

## 1 Introduction

The average maternal age at first birth has been steadily rising, reflecting both a decline in births to teenage and young adult mothers as well as an increase in first births in women in the fourth and fifth decades of life (Osterman et al., 2023). While the age at peak fecundability has been estimated to occur at 29–30 years for parous women (women who have had a prior delivery) and 27–28 years for nulliparous women (women who have never had a delivery) (Rothman et al., 2013), the birth rate for women aged 30–34 years has surpassed that of women aged 25–29 years since 2015 (Osterman et al., 2023). It is well established that the ovarian factor—the decline in oocyte quantity and quality, which occurs because of an increase in meiotic errors in this finite pool—becomes a more important consideration with age (Crawford and Steiner, 2015). Though the dogma was called into question in 2004 (Horan and Williams, 2017) when oocyte stem cells were isolated from mouse ovaries (Johnson et al., 2004), most current evidence points to the existence in human ovaries of a more or less finite, non-renewable, pool of oocytes which is gradually depleted until menopause. Therefore, given the trend towards delaying childbearing and the impact of age on ovarian reserve and fecundability, it is not surprising that the focus of scientific inquiry with respect to fertility and aging has been the ovary.

For many years, the data from assisted reproductive technology (ART) cycles aligned with the ovary-centric paradigm, and the age-related decline in fertility was ascribed mostly or entirely to ovarian aging (Navot et al., 1991; Navot et al., 1994). As a result, many technologies were developed with the aim of circumventing ovarian aging. The hurdle of ovarian aging can now be overcome via oocyte or ovarian tissue cryopreservation at a younger age, or by the use of non-autologous oocytes, though the former requires financial means and advanced planning while the latter adds financial burden and is not accepted by all patients as an alternative to the use of autologous oocytes. The use of preimplantation genetic testing for aneuploidy (PGT-A) may decrease the chance of transferring aneuploid embryos, the rates of which predictably increase with advancing age in women undergoing *in vitro* fertilization (IVF) (Franasiak et al., 2014). However, none of these technologies considers the potential impact of the age of the uterus, and more specifically the impact of aging of the endometrium (uterine mucosal lining) on pregnancy outcomes.

More recently, a reexamination of the assumptions about endometrial aging has brought the topic to the forefront. A 2022 analysis of endometrial gene expression by age revealed altered expression of genes related to cilia motility and epithelial cell proliferation in women over 35 years (Devesa-Peiro et al., 2022), identifying mechanisms that may contribute to endometrial aging. A 2023 systematic review and meta-analysis of >11,000 euploid embryo transfers found a higher ongoing pregnancy rate or live birth rate in women <35 years compared with their older counterparts (Vitagliano et al., 2023), suggesting the role of a non-ovarian factor in dictating pregnancy outcomes in older women. In an attempt to address the contribution of the endometrium to pregnancy success, the last decade has seen a rise in endometrial receptivity testing. Endometrial receptivity refers to the limited time during which the human endometrium, after undergoing morphologic and functional changes under the influence of ovarian hormones, permits embryo attachment (Achache and Revel, 2006). Endometrial receptivity testing is therefore based on the premise that the window of implantation has a distinct transcriptomic signature that should be targeted in order to personalize the timing of an embryo transfer (Ruiz-Alonso et al., 2013). This technology allowed for the characterization of the endometrium as pre-receptive, receptive, or post-receptive; it was initially considered to be a panacea in terms of correcting endometrial factor infertility, including the potential to correct for an adverse impact of age on endometrial receptivity. However, the utility of this testing has been the subject of heated debate (Quaas and Paulson, 2021; Ruiz-Alonso et al., 2021) and has not yet been assessed for applicability to women of older reproductive age (>37 years). Therefore, it is now time to reconsider the impact of age on endometrial function.

Herein we review the existing literature on endometrial aging and its impact on pregnancy outcomes. We begin by reviewing the cycle of endometrial growth, differentiation, shedding and regeneration as an overview of the principles of endometrial physiology and function. We then explore mechanisms that contribute to endometrial aging, examining each individual cellular compartment. Finally, we highlight lessons about endometrial aging that can be gleaned from studies of natural conceptions and those achieved using ART, as well as the relationship between endometrial aging and disorders of pregnancy that originate from placental dysfunction.

## 2 Endometrial physiology and function

### 2.1 Endometrial structure and function

**FIGURE 1:**
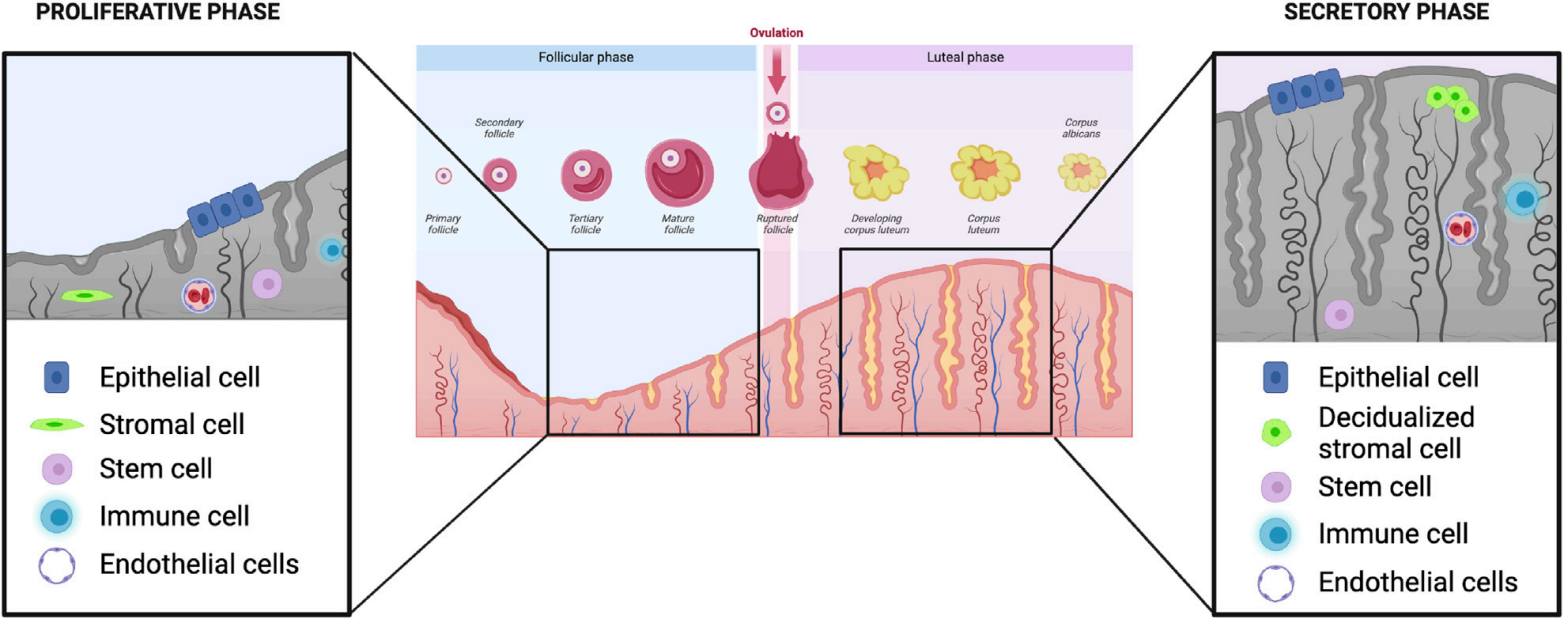
Ovarian and endometrial events in the human menstrual cycle. In the proliferative phase, estradiol secreted from the developing follicle leads to growth and development of the endometrium (left). Magnified inset shows that multiple cell types comprise the endometrium: epithelial, stromal, stem, immune and endothelial. Following ovulation, in the secretory phase, the endometrium undergoes various transformative events, including stromal cell decidualization, in preparation for possible embryo implantation (right). Images created with Biorender.com.

The endometrium is a dynamic tissue, undergoing morphologic and functional changes in response to ovarian-derived steroid hormones (Figure 1). These changes prepare the endometrium for embryo implantation, and in the absence of successful implantation and initiation of a pregnancy, the human endometrium sheds during menses and regenerates in the subsequent cycle. The endometrium is composed of two layers: the functionalis (upper two-thirds) layer, which is shed during menstruation, and the basalis (lower one-third) layer which is adjacent to the myometrium and does not shed. The functionalis layer is comprised of a columnar surface (luminal) epithelium which invaginates to form the lining of the endometrial glands, as well as stromal, vascular and immune cells. The endometrium is highly responsive to cyclic changes in the ovarian steroid hormones estradiol and progesterone, which largely exert their effects via their cognate nuclear receptors and downstream regulation of gene transcription within multiple cellular compartments of the endometrium (Jain et al., 2022).

Although both isoforms of the nuclear estrogen receptor (ER), ER-alpha and ER-beta, are expressed in all parenchymal endometrial cell types (including glandular epithelial and stromal) across all phases of the menstrual cycle, ER-alpha is the predominant form uniformly (Matsuzaki et al., 1999). ER-alpha is encoded by the *ESR1* gene on chromosome 6 and ER-beta is encoded by the *ESR2* gene on chromosome 10 (Jain et al., 2022). Progesterone receptors (PR) are encoded by a single gene on chromosome 11 and occur in two isoforms: PR-A and PR-B. PR-A is the predominant form within the endometrium. Expression of endometrial PR-A is upregulated by estrogens via ER-alpha, and thus rising estradiol levels during the proliferative phase of the menstrual cycle are required for the endometrium to respond to progesterone in the secretory phase (Yu et al., 2022).

### 2.2 Endometrial growth during the proliferative phase

Following menses, the proliferative phase of the menstrual cycle is characterized by endometrial growth and re-epithelialization largely in response to rising estradiol secreted by the dominant ovarian follicle (Figure 1, left panel). By cycle days 5–6 (where cycle day 1 is the first day of menses), the entire cavity is re-epithelialized and stromal growth begins. In the early proliferative phase, endometrial glands are narrow, tubular, and are lined by low columnar epithelial cells (Noyes et al., 1975). The blood supply to the endometrium originates from the uterine arteries, which subsequently branch into the arcuate arteries, radial arteries, and spiral arterioles which are steroid hormone-responsive vessels (Farrer-Brown et al., 1970). Throughout the proliferative phase, there is widespread cellular proliferation in all of the principal cellular compartments of the endometrium including epithelial, stromal and vascular cells; proliferation peaks on cycle days 8–10 and coincides with the highest concentration of estrogen receptors in the endometrium (Bergeron et al., 1988). During the proliferative phase, the endometrium grows, on average, from 4–5 mm–11 mm in thickness, and produces the trilaminar appearance on transvaginal ultrasound (Bakos et al., 1993).

Endometrial growth is also promoted by an increase in insulin-like growth factors (IGF) and epidermal growth factors (EGF), both of which are stimulated by rising estradiol levels during the proliferative phase (Hofmann et al., 1991; Giudice et al., 1993). Other hormones influencing endometrial growth are glucocorticoids, specifically cortisol, and androgens. Androgen receptors are predominantly expressed in the endometrial stroma during the proliferative phase with reduced expression during the secretory phase, and androgens have an anti-proliferative effect on endometrial epithelial and stromal cells (Gibson et al., 2016). Glucocorticoid receptors are expressed in human endothelial cells, and their activation in the proliferative phase reduces angiogenesis (Logie et al., 2010). Finally, Brighton et al. suggested that an organized process of senescence in, and ultimately removal of, a subgroup of endometrial stromal cells contributes to the overall remodeling and regrowth of the endometrium during the proliferative phase (Brighton et al., 2017).

Cellular senescence is a phenomenon described in proliferative tissue including the endometrium and can occur under physiologic conditions when a cell reaches its final telomere length or under the influence of oxidative stress and other stressors such as DNA damage, inflammation, and epigenetic modifications (Deryabin et al., 2020). It is especially important to distinguish the global process of aging as a clinical entity from the cellular process of senescence: cellular senescence is present throughout the lifespan and is not an inherently pathologic process. Senescent cells are present in healthy tissue, arise from replicative exhaustion, and are cleared by the immune system. In the endometrium, stromal cells differentiate during the process of decidualization into either mature decidual cells or senescent cells. These senescent cells remain metabolically active, secreting pro-inflammatory cytokines, chemokines, matrix metalloproteases and growth factors, a senescence-associated secretory phenotype (SASP) that mediates communication with the surrounding microenvironment (Coppe et al., 2008). Via secretion of SASP, senescent cells can be recognized and quickly cleared by immune cells, thus creating an appropriately regulated inflammatory environment for embryo implantation (Deryabin et al., 2020).

While serving a physiologic function, senescence may also be associated with pathological conditions such as neurodegeneration and atherosclerosis, cancer and immune system dysregulation. Many of these conditions are “age-related” in that damage is accrued over time. And indeed, during natural aging, immune clearance of senescent cells in the endometrium may be impaired. These senescent cells are not able to decidualize properly and their secretory phenotype can create a pro-inflammatory microenvironment that may interfere with endometrial receptivity (van Deursen, 2014; Deryabin et al., 2020). To this end, the elimination of accumulated senescent cells is currently being investigated as a strategy to alleviate various age-related diseases and tissue malfunction (Baker et al., 2011; Zhu et al., 2015) and may have future applications to the endometrium.

### 2.3 Endometrial changes and remodeling during the secretory phase

Whereas during the proliferative phase the endometrium is mostly responsive to estradiol alone, after ovulation, in the secretory phase (Figure 1, right panel), the endometrium responds to both estradiol and progesterone secreted by the ovarian corpus luteum formed post-ovulation. During the secretory phase, the total endometrial thickness remains relatively fixed, despite continued elevated estradiol levels (Bakos et al., 1993). This is at least partly attributable to the action of progesterone, which limits growth of the endometrium via decreased mitosis and DNA synthesis (Moyer and Felix, 1998). Furthermore, progesterone stimulates the activity of 17beta-hydroxysteroid dehydrogenase and sulfotransferase, which converts estradiol to estrone sulfate, the form of estrogen that is more rapidly cleared from cells (Gurpide et al., 1976; Falany and Falany, 1996). Finally, progesterone antagonizes estrogen-mediated stimulation of oncogenes, which would otherwise promote further endometrial growth (Kirkland et al., 1992). Progesterone receptor expression in endometrial cells is stimulated by increased circulating estradiol during the proliferative phase via ER-alpha. In a negative feedback loop, further ER-alpha receptor expression in endometrial cells is inhibited by progesterone (West and Brenner, 1985). While PR-A levels start to decline, PR-B expression levels remain constant during the secretory phase and PR-B plays a role in the control of glandular secretion. In a tightly controlled feedback loop, progesterone then limits expression of ER-alpha via PR-A (McDonnell and Goldman, 1994). This feedback loop is essential for creating the specific microenvironment that allows for endometrial response to estrogen in the proliferative phase, while simultaneously preventing an over-response, leading to endometrial hyperplasia.

As the endometrium prepares for the possibility of embryo implantation it undergoes a process of remodeling that includes functional and morphological changes. Approximately 7-8 days after ovulation, circulating levels of estradiol and progesterone promote an increase in circulating prostaglandins and VEGF. These factors influence capillary permeability, thereby resulting in stromal edema, a hallmark of this phase of the cycle (Plaisier, 2011). Rising progesterone triggers a marked increase in multiple leukocyte populations, which ultimately comprise up to 40% of all endometrial cells (King, 2000). These leukocytes serve multiple roles, including immunoprotection as the endometrium prepares for implantation, regulation of trophoblast invasion, and in the absence of pregnancy, breakdown of endometrial tissue in the menstrual phase via secretion of matrix metalloproteinases (Mihm et al., 2011). Towards the end of the secretory phase, prior to the onset of menses, the endometrium has differentiated into three zones: the deepest layer remains the unchanged basalis layer, the middle layer is the stratum spongiosum, or loose edematous stroma containing tightly coiled spiral vessels and dilated glands, and the most superficial layer is the more stromally dense stratum compactum (Heremans et al., 2021). A hallmark of endometrial remodeling in the secretory phase is the transition of endometrial stromal fibroblasts into epithelioid-like decidualized stromal cells, characterized by increasing amounts of glycogen and lipids, and an expanded cytoplasm (Owusu-Akyaw et al., 2019). Decidualized stromal cells secrete both prolactin and insulin-like growth factor binding protein (IGFBP-1), which stimulate trophoblast growth and invasion, along with other actions that together produce the morphologic changes that aid embryo implantation (Owusu-Akyaw et al., 2019). The degree to which this process is completed optimally results in endometrial receptivity, which refers to the preparation of the endometrium for embryo implantation during the specific 4–6 day “window of implantation” in the secretory phase (Lessey and Young, 2019).

### 2.4 Endometrial shedding

In the absence of pregnancy, demise of the corpus luteum leads to withdrawal of estradiol and progesterone, initiating the process by which two-thirds of the endometrium is shed during menstruation. Immediately after hormone withdrawal, the tissue height (particularly within the upper zone of the functionalis layer or the stratum compactum) decreases. The reduction in blood flow within spiral arterioles triggers vasoconstriction which causes local ischemia. Menstruation occurs due to resulting endometrial ischemia as well as enzymatic degradation from the release of lysosomes containing lytic enzymes, which promote apoptosis in both glandular epithelial and stromal cells (Rosenwaks and Seegar-Jones, 1980; Ferenczy, 2003; Jain et al., 2022).

### 2.5 Endometrial regeneration

The process of endometrial regeneration originates, at least in part, from epithelial and stromal stem/progenitor cell populations residing in the basalis layer (Salamonsen et al., 2021). Re-epithelialization of the exposed surface of the endometrium is accomplished by endometrial epithelial stem cells located in glands within the basalis layer (Cousins et al., 2021). Circulating estradiol, secreted by a new dominant ovarian follicle, begins to increase following menses, prompting the glandular proliferation necessary for regenerating the functionalis layer (Gargett et al., 2016). By days 2–3 of the cycle, DNA synthesis occurs in the areas of the basalis that have now been exposed due to menses. Garry et al. observed that menstrual shedding occurs in a piecemeal manner, with areas of shedding and healing endometrium existing simultaneously (Garry et al., 2009). The regeneration process is supported by stromal fibroblasts which direct regrowth of the endometrium via autocrine and paracrine mechanisms; endometrial stromal cells contribute to the regeneration not only of the stromal compartment but also the epithelial compartments via mesenchymal-epithelial transition (Owusu-Akyaw et al., 2019). The repair processes occurs rapidly; new epithelium covers more than two-thirds of the endometrial cavity by cycle day 4. The repair process is also, at least in part, likely a response to injury and inflammation caused by high levels of circulating cytokines and proteolytic enzymes (Salamonsen et al., 2021).

## 3 The aging of individual cell types in the human endometrium

### 3.1 Histopathologic changes

There are few reports that describe how the clinical characteristics of the uterus and endometrium change with age. Uterine size and volume are known to decrease following menopause (Merz et al., 1996). In a recent study of 146 Chinese women, Li et al. (Li et al., 2020) found that the mean uterine volume decreased from 44.9 ± 17.8 cm^3^ prior to menopause, to 16.3 ± 10.6 cm^3^ after menopause; mean endometrial thickness decreased from 4.8 ± 2.1 to 3.3 ± 0.5 mm. In a sub-analysis of patients that did not have fibroids, uterine volume was noted to decrease even prior to the final menstrual period (Li et al., 2020). Histologically, the aging endometrium has been characterized as inactive in the decade after menopause, to senescent or atrophic thereafter (Ferenczy and Bergeron, 1991). This distinction is used to differentiate between a tissue without mitotic activity but whose cells contain estrogen receptors and are able to respond to hormonal stimulation (inactive), and cells that are atrophic and no longer express estrogen receptors (severely atrophic) (Ferenczy and Bergeron, 1991). However, in order to better understand the cellular changes occurring in the endometrium over the reproductive and post-reproductive lifespan, the individual cellular compartments (stromal, epithelial, vascular, immune and stem cells), and their role with respect to embryo implantation, must be examined (summarized in Table 1).

**TABLE 1:** Age-related changes in individual cellular compartments.

Cell type	Cell function	Changes with age
Stromal cells	Undergo decidualization following exposure to progesterone; decidualized stromal cells secrete peptide hormones and growth factors that stimulate trophoblast growth and invasion	- Increased cellular senescence associated with poorer pregnancy outcomes (Tomari et al., 2020)
		- Differential gene regulation, particularly *PR* and *ESR1/2* (Erikson et al., 2017)
		- Decreased proliferation (Berdiaki et al., 2022)
		- Decreased expression of decidualization markers (Berdiaki et al., 2022)
Luminal epithelial cells	Represents the first point of contact with the implanting embryo	- Increased p16 and p21 expression (Lv et al., 2022)
		- Increased expression of profibrotic *PTGS2* (Lv et al., 2022)
		- Decreased expression of cell cycle related gene *PCNA* (Lv et al., 2022)
		- Upregulation of NF-kappa B signaling pathway and extracellular matrix receptor interaction associated with cellular senescence (Lv et al., 2022)
		- Lower secretion of bactericidal substances (e.g., SLPI) in post-menopausal women (Fahey and Wira, 2002)
Glandular epithelial cells	Secretory products signal to embryo and provide fetus with nutrition during organogenesis	Increased somatic mutation burden (Moore et al., 2020)
Endothelial cells	Express steroid hormone receptors, undergo remodeling in response to ovarian steroid hormones	- Diminished vasoreactivity in post-menopausal uterine arteries (Nicholson et al., 2017)
		- Increased myometrial artery calcifications (Hessler et al., 2015)
Immune cells	Protect against infection, while creating an environment that is not hostile to allogenic sperm and permissive of embryo implantation	- Higher cytolytic potential in CD3^+^ T cells in post-menopausal subjects (White et al., 1997)
		- Increased cytotoxic activity of CD8^+^ T cells after menopause (Rodriguez-Garcia et al., 2020)
		- Higher frequency of Th17, CD8^+^ T cells, and CD8^+^ tissue-resident memory T cells after menopause (Rodriguez-Garcia et al., 2014; Rodriguez-Garcia et al., 2018)
Stem cells	Contribute to endometrial regeneration and serve immunomodulatory functions in endometrium	Reduced expression of SHH and increased expression of SERPINB2 (Cho et al., 2019)

SLPI, secretory leukocyte protease inhibitor; SHH, sonic hedgehog.

### 3.2 Stromal cells

Endometrial stromal fibroblasts, mesenchymal in origin, proliferate during the proliferative phase of the menstrual cycle and undergo decidualization following exposure to progesterone after ovulation. Stromal cells depart the cell cycle to differentiate into decidualized cells, a process mediated by transcription factors including C/EBPs, FOXO1, CREB and STAT5 (Deryabin et al., 2020). Decidualized endometrial stromal cells secrete a variety of peptide hormones and growth factors, including prolactin, relaxin, renin, insulin-like growth factors (IGFs) and insulin-like growth factor-binding proteins (IGFBPs) (Fritz and Speroff, 2011). Senescence, the point at which mitotic activity ceases in normal cells, can be measured by specific markers including p21 and senescence-associated β-galactosidase (SA-β-Gal). Cellular senescence is a physiologic process that can also occur after exposure to non-physiologic stressors (Deryabin et al., 2020).

Senescence of endometrial fibroblasts has been associated with poor reproductive outcomes. Tomari et al. examined the impact of stromal cell senescence on endometrial receptivity, using endometrial biopsies taken at the time of oocyte retrieval, 2 days prior to embryo transfer into the uterus (Tomari et al., 2020). Patients with implantation failure (i.e., who did not achieve pregnancy after embryo transfer) were classified as having a non-receptive endometrium. β-galactosidase activity, a marker of cellular senescence, was found to be increased in human endometrial stromal cells (HESCs) isolated from non-receptive endometrium as compared to HESCs isolated from receptive endometrium. Cell cycle arrest is a hallmark of senescent cells (Ogrodnik et al., 2019); whereas HESCs isolated from women who achieved pregnancy were more frequently in S phase (actively replicating), HESCs isolated from subjects with implantation failure were more frequently in G0/G1 phase, suggesting senescent cells in cell cycle arrest. Cellular senescence-related genes *CDKN1A* and *CDKN2A* were also more highly expressed in ESCs isolated from the endometrium of subjects with implantation failure (Tomari et al., 2020). The results of this study, demonstrating an inverse association between HESC senescence and embryo implantation, suggest that implantation failure may be attributed, at least in part, to aging of HESCs.

In addition to assessing markers of stromal cell senescence, other methods have been used to show the impact of age on endometrial stromal cells. In a transcriptomic analysis of pre-menopausal and peri-menopausal stromal fibroblasts, over 1,000 genes were found to be differentially expressed between these two age groups (Erikson et al., 2017). Ingenuity pathway analysis demonstrated the pathways of interest to be related to cell-cycle processes (“organization of cytoskeleton”, “formation of filaments” “formation of actin stress fibers”), as well as to fibroblasts (“proliferation of fibroblasts”, “migration of fibroblast cell lines”), and identified differential regulation of additional pathways common with senescent or aging cells. Quantitative real-time PCR validation of specific genes revealed downregulation of both the progesterone receptor (*PR*) and estrogen receptor beta (*ESR2*), and upregulation of estrogen receptor alpha (*ESR1*) in the younger cohort (Erikson et al., 2017). This study also examined the transcriptomic profile of endometrial mesenchymal stem cells (eMSCs) in these two age groups and performed a four-way comparison (pre- and peri-menopausal stromal fibroblasts, and pre- and peri-menopausal eMSCs). They found that the gene expression profile of perimenopausal stromal fibroblasts more closely resembled that of the eMSC groups, suggesting that the perimenopausal stromal fibroblasts are less differentiated than premenopausal stromal fibroblasts (Erikson et al., 2017).

*In vitro* studies have demonstrated that hypoxia-induced endometrial stromal cell senescence leads to impaired decidualization as evidenced by incomplete morphological transformation and reduced expression of decidualization markers *IGFBP1* and *PRL* (Deryabin and Borodkina, 2022). Kusama et al. found that *in vitro* decidualization of HESCs, confirmed by upregulated expression of *PRL*, *IGFBP1* and *FOXO1*, resulted in an increase in expression of senescence markers *p21* and *p53*, suggesting that decidualization may promote cellular senescence (Kusama et al., 2021). Treatment of decidualized HESCs with Dasatinib and Quercetin, which have been extensively studied in combination as a senolytic treatment due to their selective clearance of senescent cells (Xu et al., 2018; Islam et al., 2023), increased expression of decidualization markers such as *PRL* and decreased the number of senescent cells (Kusama et al., 2021).

In a study of endometrial stromal cells from women aged 25–46 years, endometrial biopsies were taken in the proliferative phase and proliferation was assessed using a fluorometric assay. Women aged 25–35 years had significantly higher levels of stromal cell proliferation compared with women aged 36–46 years. Women in the younger age group had significantly higher expression of BMP2 and STAT3, two important regulators of stromal cell proliferation and differentiation. Following *in vitro* decidualization, endometrial stromal cells from younger women also had significantly higher expression of PRL and IGFBP-1, two key markers of decidualization (Berdiaki et al., 2022). Collectively these data implicate aberrations in stromal cell functions during the process of endometrial aging, including changes in cell cycle regulation, decreased proliferative capacity and impaired decidualization, as well as alterations in the transcriptomic profiles of stromal fibroblasts to resemble stem cells more closely. These age-related changes may be at least partially mediated by the altered expression of steroid hormone receptors PR and ESR1 and ESR2.

### 3.3 Epithelial cells

The epithelial cells of the endometrium can be subdivided into those lining the endometrial glands (glandular epithelium) and those lining the lumen (luminal epithelium). The luminal epithelial cells are the first point of contact with the implanting embryo; the secretory products of glandular epithelial cells signal to the embryo and provide the fetus with nutrition during organogenesis (Burton et al., 2002). Epithelial cells, too, have been investigated with respect to their role in endometrial aging.

A recent study linked accelerated cellular senescence in epithelial cells with inadequate endometrial development, a clinical condition known as “thin endometrium” that is associated with decreased clinical pregnancy and live birth rates. While an association between thin endometrium and age has not yet been established, this study points to a possible link between the two entities. The authors explored important changes in the luminal epithelium of subjects with thin (<7 mm) compared with normal (8–14 mm) endometrium, using single cell RNA sequencing on endometrial biopsy samples taken in the late proliferative phase of a natural menstrual cycle in women who presented for evaluation to a fertility clinic (Lv et al., 2022). The authors found that despite having similar cell types, thin endometria had decreased numbers of proliferating stromal cells relative to normal endometria, as well as decreased luminal epithelium with relatively increased glandular epithelium. They additionally found increased immunostaining for p16 and p21, markers of cellular senescence, as well as changes in gene expression in thin endometria, such as increased expression of the profibrotic enzyme PTGS2 (COX-2) and decreased expression of cell cycle-related gene PCNA; these aberrations are suggestive of excessive collagen deposition. Finally, they report changes in signaling pathways, such as upregulation of NF-kappa B signaling and extracellular matrix receptor interaction pathways in thin endometria. Taken together, these findings suggest an endometrial phenotype characterized by accelerated cellular senescence and increased fibrosis in the epithelial cells of thin endometria (Lv et al., 2022).

In addition to their role as the first point of contact with an implanting embryo, luminal epithelial cells play a crucial role in protecting the endometrium against pathogens via tight junctions to regulate passage of molecules across the barrier. The permeability of this layer is mediated, at least in part, by fluctuations in estradiol (Wira et al., 2015). Luminal epithelial cells also exhibit immune properties which change with aging; luminal epithelial cells secrete substances such as secretory leukocyte protease inhibitor (SLPI), a protein with bactericidal properties which has been shown to be higher concentrations in premenopausal as compared to postmenopausal. In this study, pre-menopausal endometrial epithelial cells were found to have greater ability to kill *Staphylococcus aureus* and *Escherichia coli* in culture than endometrial cells from post-menopausal women and this correlated directly with SLPI levels (Fahey and Wira, 2002).

The glandular epithelium has been implicated in endometrial aging as well. A study of the somatic burden of endometrial glands of women aged 19–81 years found that approximately 29 base substitutions were added per gland per year, without a correlation to parity (Moore et al., 2020). Together, these studies suggest that epithelial cell senescence may be the consequence of alterations in proliferation, changes in the composition of the microenvironment, and increasing mutational burden.

### 3.4 Vascular cells

Each layer of the endometrium is supplied by a network of vessels originating from the uterine arteries which course through the myometrium: the basal arteries supply the basalis layer, spiral arterioles feed the functionalis layer, and the subepithelial capillary plexus supports the subepithelial zone (Massri et al., 2023). Human endometrial vasculature undergoes cyclic remodeling under the influence of estrogen in the proliferative phase and progesterone in the secretory phase. Endometrial endothelial cells express estrogen and progesterone receptors and undergo remodeling in response to ovarian steroid hormones, as well as in response to angiogenic factors such as VEGF and FGF2 secreted by epithelial and stromal cells (Massri et al., 2023).

Mechanisms governing aging of the endometrial vasculature can only be inferred at best, based on studies demonstrating adverse effects of age on uterine artery function. The vasoreactivity of the uterine arteries was examined in hysterectomy samples of pre- and post-menopausal subjects, and menopause was found to be associated with diminished vessel constriction and relaxation, as well as an impairment in the vasodilatory response with exposure to 17β-estradiol (Nicholson et al., 2017). In a separate study, myometrial artery calcifications were evaluated in women who had undergone hysterectomy. No calcifications were observed in women aged 45–49 years, and the rate of myometrial artery calcification increased with increasing age with 50% of women aged 70–81 years demonstrating calcifications (Hessler et al., 2015). Although these studies examine uterine artery aging, it is plausible that the processes reported in these larger vessels are occurring in the smaller endometrial vessels derived from them, and even that aging in larger vessels may impact the vessels that branch from them.

### 3.5 Immune cells

The immune cells in the human endometrium have the dual role of protecting against ascending infections, while creating an environment that is not hostile to allogenic sperm and is permissive of embryo implantation (Wira et al., 2015). Endometrial immune cell populations must be responsive to sex steroids (Shen et al., 2021), and indeed, multiple studies have confirmed the suppressive effects of estradiol on endometrial T cells (Nilsson and Carlsten, 1994; Rijhsinghani et al., 1996; White et al., 1997; Rodriguez-Garcia et al., 2020; Shen et al., 2021). T cells, key mediators of the adaptive immune system, are present in the endometrium and their populations change with age. In an early study of T cell function in the endometrium in pre- and post-menopausal subjects, CD3^+^ T cells were found to have higher cytolytic potential in the proliferative phase compared with the secretory phase of pre-menopausal subjects, and highest cytolytic potential was found in post-menopausal subjects, suggesting that the cytolytic activity is suppressed in preparation for embryo implantation in a hormone-mediated manner and that this function is lost after menopause (White et al., 1997). More recent studies have found that pre-treatment of CD8^+^ T cells with estradiol at physiologic concentrations, followed by co-culture with allogenic target cells, reduced the number of dead target cells relative to co-culture of untreated T cells and target cells suggesting an estradiol-mediated reduction in T cell cytotoxicity (Shen et al., 2021).

Other studies have found increased cytotoxic activity of CD8^+^ T cells (Rodriguez-Garcia et al., 2020) and changes in T cell populations following menopause: the frequencies of Th17 and CD8^+^ T cells were found to be increased in menopausal endometrium (Rodriguez-Garcia et al., 2014; Rodriguez-Garcia et al., 2018) and CD8^+^ tissue-resident memory T cells, identified as CD103^+^, have been found in greater abundance in postmenopausal endometrial samples (Rodriguez-Garcia et al., 2018). Collectively, these studies point to an estrogen-dependent suppression of endometrial T cells which becomes less robust with the age-related decline in estrogen concentrations.

### 3.6 Stem cells

The lower basalis layer serves as the source for cyclic regeneration of the endometrium (Salamonsen et al., 2021). Given that the regenerated tissue is composed of various cell types, the origin, location and characteristics of endometrial stem cells are of great interest (Gargett et al., 2016). Epithelial stem/progenitor cells and mesenchymal stem cells (eMSC) have been isolated from human and rodent endometrium (Bozorgmehr et al., 2020; Cousins et al., 2021) and have been shown to have classic properties of stem cells, including clonogenicity and in the case of eMSC, multilineage differentiation potential (Gargett et al., 2009).

Stem cells play an important role in the maintenance, repair or regeneration of nearly all tissues in the human body. These cells reside in various organs in a quiescent state and are called upon to differentiate in response to growth or injury (Jung and Brack, 2014). However, stem cells are susceptible to age-related damage that impairs their regenerative capacity (Liu et al., 2022). Certain tissues, such as the intestinal mucosa, undergo frequent regeneration similar to the endometrium, and these stem cells, too, are susceptible to aging. Intestinal stem cells which are found at the base of the intestinal crypts, bear some similarities to endometrial epithelial stem/progenitor cells that reside in the basalis layer of the endometrium and contribute to the repair and re-epithelialization that takes places following menses (Cousins et al., 2021). Endometrial epithelial stem/progenitor cells and intestinal stem cells share certain properties—namely that they are tissue resident, localized to the deeper layers (basalis layer of the endometrium and intestinal crypts, respectively), and permit the tissue to undergo constant renewal (Baulies et al., 2020; Bozorgmehr et al., 2020). Thus, though studies describing the impact of aging on endometrial stem cells are limited at present, findings in the intestine may provide relevant insights. Age-related changes in intestinal stem cells are reflected in the decreased number of crypts, increased crypt length and width, and fewer numbers of proliferating stem cells noted with aging (Liu et al., 2022). In murine studies, intestinal stem cells in aged mice exhibited functional impairment, with decreased migration capacity from crypt to villus (Nalapareddy et al., 2017).

In the endometrium, several studies have explored the molecular changes that stem cells incur with age, in order to better understand the implications for reproductive health. One such study (Cho et al., 2019) examined the sonic hedgehog (SHH) protein, which functions principally as a morphogen during embryonic development but has also been found to play a role in age-related conditions such as neurodegenerative disease and atherosclerosis (Queiroz et al., 2012; Cho et al., 2019). In this study, endometrial tissues from aging mice were found to express significantly lower levels of SHH mRNA and protein compared to young mice. Treatment of human endometrial mesenchymal stem cells with SHH alleviated senescence-induced dysfunctions, improving self-renewal, migratory, and multilineage differentiation capacities. These effects, mediated by downregulation of SERPINB2, provided new insights into the mechanisms regulating endometrial stem cell aging, and identified SERPINB2 as a potential biomarker and therapeutic target in aging endometrial stem cells (Cho et al., 2019).

Endometrial stem cell dysfunction has been implicated in adverse pregnancy outcomes such as recurrent pregnancy loss (RPL), a clinical entity that is more common with advancing female reproductive age (Magnus et al., 2019). W5C5 is a marker of perivascular endometrial mesenchymal stem cells (eMSCs); W5C5- eMSCs can self-renew, demonstrate multilineage differentiation, and can reconstitute mesodermal tissue (Masuda et al., 2012). In a study of secretory phase endometrial biopsies, subjects with RPL were found to have reduced clonogenicity in both W5C5+ (perivascular) and W5C5- (non-perivascular) stromal cells (the latter representing a heterogenous group of non-stem cells such as stromal, vascular or immune cell types) relative to controls (Lucas et al., 2016). In a study of secretory phase endometrial biopsies, subjects with RPL were found to have reduced clonogenicity in both W5C5+ (perivascular) and W5C5- (non-perivascular) stromal cells relative to controls (Lucas et al., 2016). Further, the number of clonogenic cells demonstrated a negative correlation with the number of miscarriages (Lucas et al., 2016). Subjects with RPL were also found to have hypomethylation of the *HMGB2* gene, a marker of reproductive senescence in stromal cells, associated with reduced potential for proliferation and diminished endometrial plasticity (Lucas et al., 2016). These studies suggest that increasing miscarriage risk in older women may not be due to embryo aneuploidy alone, and imply a role for aging eMSC and other endometrial cell types; but proof of these concepts requires additional study.

In addition to tissue regeneration, mesenchymal stem cells also serve immunomodulatory functions; MSCs have been shown to inhibit the functions of the innate and adaptive immune systems by impairing dendritic cell function, diminishing natural killer cell cytotoxicity, and preventing T cell differentiation (Sotiropoulou et al., 2006; Willis et al., 2018; He et al., 2019; Lee et al., 2021). In the endometrium, MSCs exert paracrine effects on their neighboring stromal cells, as evidenced by *in vitro* studies in which endometrial stromal cells co-cultured with MSCs demonstrate increased proliferation, migration and invasion relative to controls (Zhao Q. et al., 2023). In the context of endometrial aging, it is interesting to note that MSCs with clonogenic activity have even been isolated from inactive post-menopausal endometrium and appear to have similar self-renewal capacity to MSCs isolated from premenopausal endometrium (Schwab et al., 2005; Ulrich et al., 2014). However, whether MSCs isolated from postmenopausal endometrium retain their paracrine/immunomodulatory effects on neighboring endometrial cells, and/or capacity to regenerate tissue *in vivo*, has not been proven. Given the crucial role of these stem cells in regenerating the endometrium and maintaining its principal functions, and given their presence throughout the lifespan, the question of their role in regulating endometrial aging is particularly interesting and should be examined in future studies.

## 4 Insights from rodent studies

**FIGURE 2:**
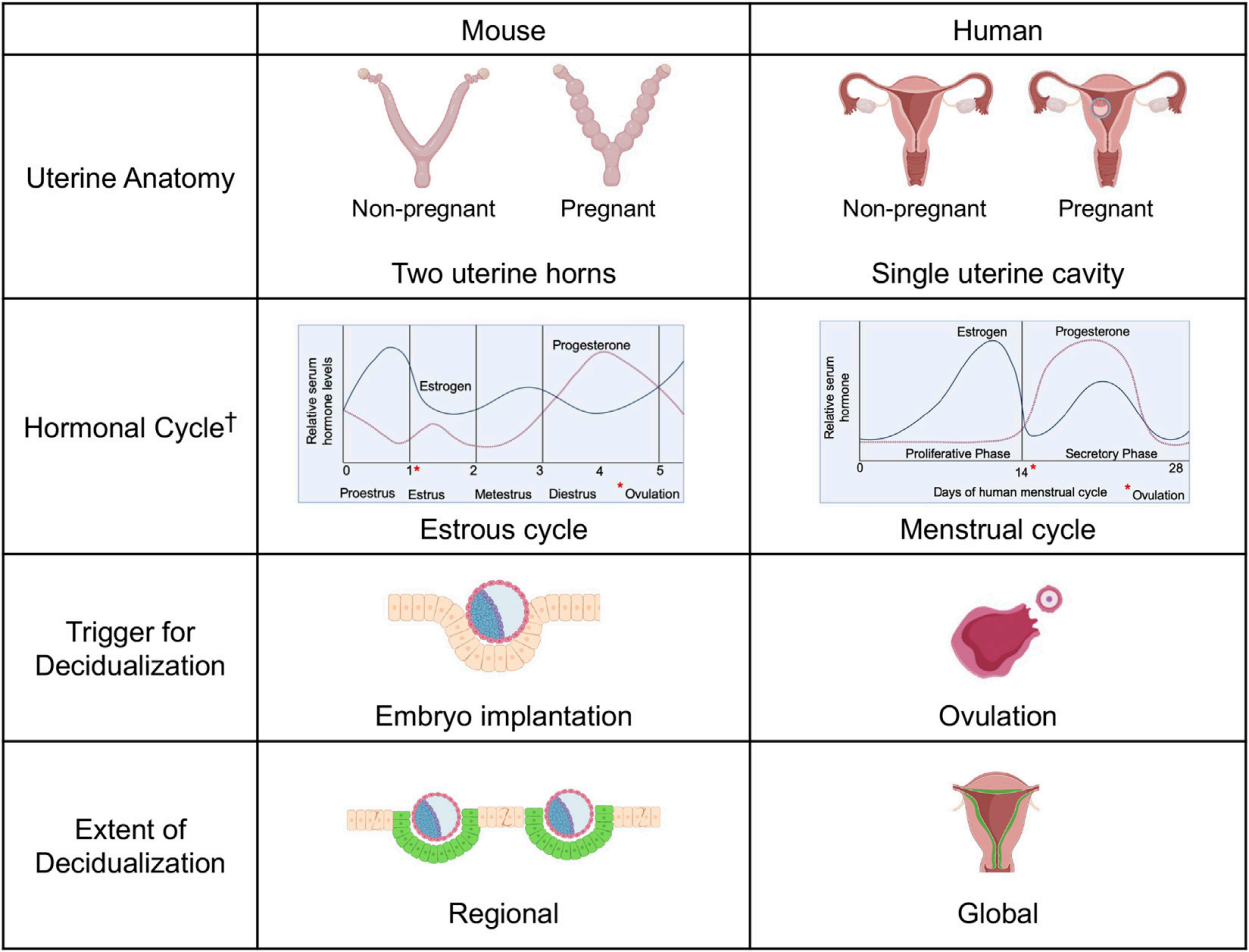
Mouse and Human Comparative Reproductive Anatomy and Physiology. Female mouse uteri contain two horns, while human uteri generally have a single cavity. The mouse estrous cycle is composed of proestrus, estrus, metestrus and diestrus stages, with ovulation marking the transition from proestrus to estrus. The human menstrual cycle is divided into the proliferative and secretory phases, with ovulation marking the transition between the two. In the mouse, the trigger for stromal cell decidualization is embryo implantation, after which stromal cells undergo mesenchymal to epithelial transition and adopt the decidualized phenotype. In the human, the trigger for decidualization is ovulation. Images created with Biorender.com. ^†^Reprinted from “Vascular changes in the cycling and early pregnant uterus” by Massri N, Loia R, Sones JL, Arora R, Douglas NC, 2023, JCI Insight. 2023 June 8; 8 (11):e163422. Copyright [2023] by the American Society for Clinical Investigation. Reprinted with permission.

Because of similarities between rodents and humans, studies in rodent models have contributed greatly to our understanding of human pathophysiology. While human reproductive anatomy and physiology overlaps substantially with the rodent, certain key morphologic and functional differences between the two species should be noted. These include differences in anatomy, lack of menstruation in rodents, cycle length, timing of ovulation and trigger for and extent of decidualization. These important distinctions are summarized in Figure 2. Despite these differences, rodent studies remain crucial to the study of human disease as they contribute *in vivo* mechanistic data (for example, knockout models) that cannot be obtained from human studies.

To determine whether aging impacts the response of the endometrium to estradiol, Bader et al. (Bader et al., 2012) employed aromatase knockout mice (ArKO) as a model of estrogen deficiency. Markers of estrogen response in the uterus, including lactoferrin (*Ltf*), estrogen receptor alpha (*Esr1*), and estrogen receptor beta (*Esr2*), were measured. Exposure of young adult (3 months) and aged adult female mice (12 months) to estradiol resulted in an age-dependent response with significantly higher expression of *Esr1* in young adult mice. While *Ltf* expression was found to increase in both age groups of mice after estradiol exposure, the effect was ten times more pronounced in young adult animals. Together, these changes suggest an age-dependent reduction in endometrial responsiveness to estrogen.

To explore the effect of steroid hormones on uterine expression of estrogen-responsive genes *Ltf* and *C3*, and progesterone-responsive genes *Ihh, Lif, Areg, Hoxa10* and *Hand2*, Li et al. (Li et al., 2017) studied ovariectomized mice between 2- and 12-months-old supplemented with subcutaneous estradiol or progesterone. They found no difference between young and aged mice in expression of *Ltf* and *C3* after treatment with estradiol; however, *Ltf* expression was lower in aged mice following progesterone treatment. Expression of progesterone-induced implantation-related genes *Lif* and *Hand2* as well as *Indian hedgehog (Ihh),* a gene involved in mediating the communication between the endometrial epithelium and stroma required for embryo implantation, was reduced in aged mice. The effects of age were also assessed by the detection of telomerase activity with real-time PCR after exposure to steroid hormones. Telomerase is an enzyme that prevents shortening of telomeres, and reduced expression may suggest diminished protection from telomere shortening and cellular senescence. In this study, 12-month-old mice had significantly lower levels of telomerase expression compared to 2-month-old mice after exposure to progesterone. Overall, this study suggests that the age-related decline in fertility may be associated with an alteration in endometrial responsiveness to progesterone, as well as decreased telomerase activity.

Cellular senescence may also contribute to aging of the rodent endometrium. To explore age-related changes in genes and pathways related to cellular senescence, Kim et al. (Kim and You, 2022) studied mRNA and microRNA expression from uterine tissue of young (3 months) and old (11 months) mice in the metestrus phase of the estrous cycle. More than 500 differentially expressed genes were identified; pathway analysis revealed changes in cellular senescence-associated pathways including arachidonic acid and glutathione metabolism. Seven microRNAs were identified as interacting with differentially expressed genes in relevant pathways such as arachidonic acid and glutathione metabolism, suggesting a role in regulating endometrial aging. Furthermore, administration of the traditional herbal medicine Samul-tang to the aged mice reduced expression of one of these microRNAs (miR-223–3p), identifying potential therapeutic targets for regulating cellular senescence in the endometrium. Taken together, rodent studies have provided some insight into the age-related changes in the endometrium—such as increase senescence and diminished hormone responsiveness—which may partially explain some of the mechanisms behind the observed age-related decline in fertility.

## 5 Insights from human clinical studies

**FIGURE 3:**
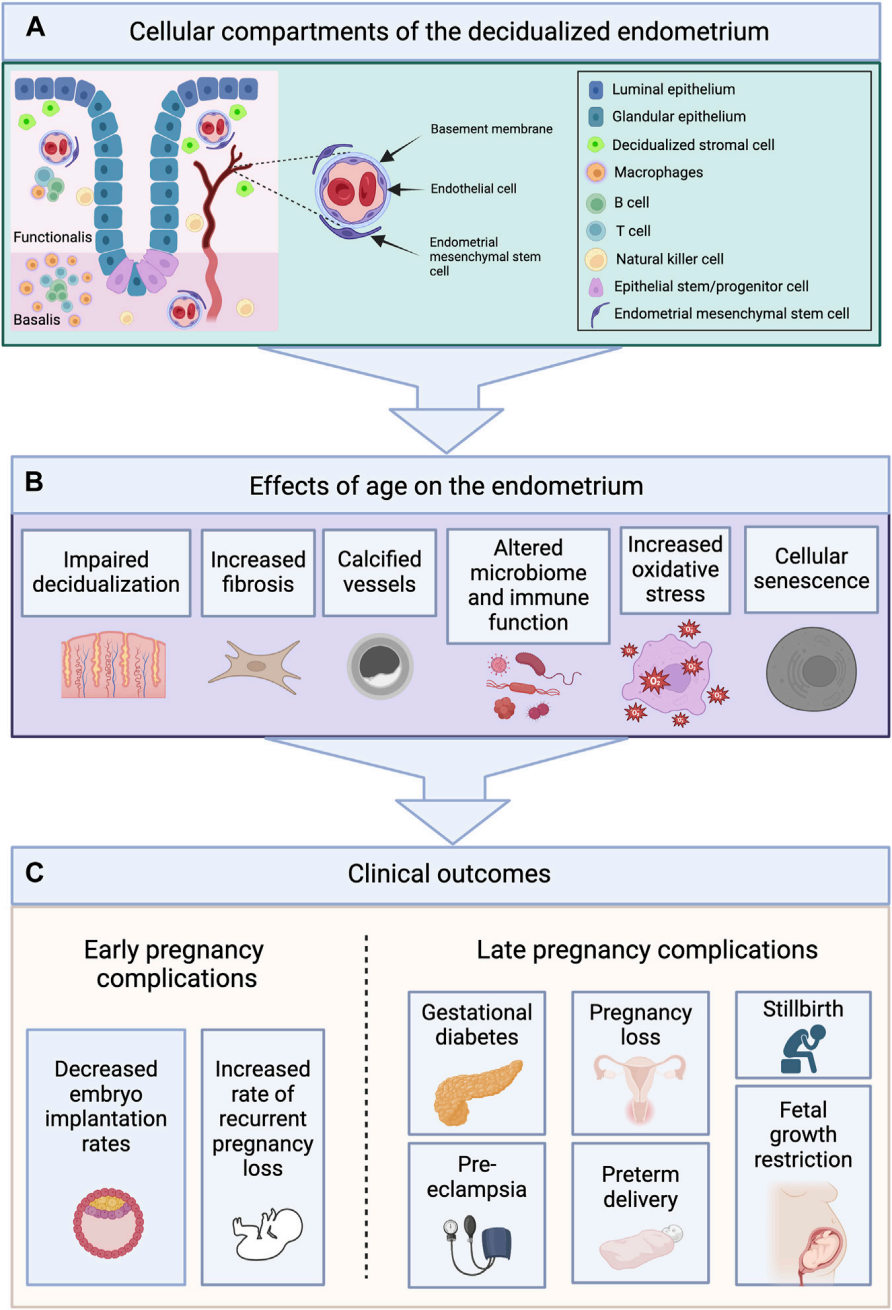
Cellular Compartments of the Endometrium Undergo Age-Related Changes Which May Contribute to Adverse Pregnancy Outcomes. **(A)** The endometrium is composed of two layers—the functionalis (upper two-thirds), and the basalis (lower one-third)—and contains various cellular compartments including epithelial, stromal, vascular and immune. **(B)** The cellular compartments of the endometrium undergo age-related changes including impaired decidualization, increased fibrosis, vascular calcification, alterations in the microbiome and immune cell populations, increased oxidative stress, and cellular senescence. **(C)** These changes may contribute to the early and late pregnancy complications observed with age. Images created with Biorender.com.

Along with rodent studies, clinical studies have been designed to address the question of endometrial aging as well (summarized in Figure 3 and Table 2). In women, the decline in fertility typically begins around age 30 and becomes clinically significant between the ages of 35 and 40 years, after which there is an even greater decrease (Menken et al., 1986). In addition to the well-established ovarian contribution to reproductive aging, the endometrium may play an important role; indeed, this tissue may be responsible for as many as two-thirds of known implantation failures and may be involved in the IVF-associated clinical entity of recurrent implantation failure, defined as women who have had three failed transfers of good-quality embryos (Bashiri et al., 2018; Craciunas et al., 2019).

**TABLE 2:** Age-related changes in the endometrium: human studies.

Biological process	Changes with age	Study limitations	References
Endometrial receptivity	Differential gene expression	Pregnancy outcomes not assessed	Devesa-Peiro et al. (2022)
DNA methylation	Increased DNA methylation in the endometrium	Clinical outcomes not assessed	Olesen et al. (2018)
Endometrial microbiota	Increased rates of endometrial dysbiosis with advancing age	Pregnancy outcomes not assessed	Fujii and Oguchi (2023)
Oxidative stress	Oxidative stress may increase with age, estrogen may have protective effects	Few studies, not a well-established effect	Kaltsas et al. (2023)
Pregnancy after IVF	Lower live birth rates in older women undergoing transfer of euploid embryos derived from autologous oocytes	Lower LBRs may be attributable to unidentified age-related changes in the embryo other than ploidy	Vitagliano et al. (2023)
	Lower live birth rate and higher miscarriage rate in older women undergoing transfer of embryos derived from donor oocytes	Variable definitions of advanced age of embryo recipients	Zhao et al. (2023b)
	Lower embryo implantation rates in women with evidence of premature endometrial aging	Premature aging of the endometrium is not a well-established entity	Shapiro et al. (2016)

IVF, *in vitro* fertilization; LBR, live birth rate.

However, until recently, the role of the endometrium in reproductive aging had been underestimated and understudied. Early studies emphasized ovarian aging as the predominant factor in predicting IVF outcomes in women of advanced reproductive age. Navot et al. (Navot et al., 1991) compared IVF outcomes in infertile women aged ≥40 years who underwent IVF using autologous oocytes or oocytes from younger donors and unsurprisingly found higher implantation, pregnancy and delivery rates in recipients of oocyte donation compared to women undergoing IVF with autologous oocytes, underscoring the importance of oocyte quality. Since this study was not able to isolate the contribution of the endometrium, a subsequent prospective study by the same group was undertaken (Navot et al., 1994). In this second study, donor oocyte-derived embryos from young women with a mean age 30.2 years were transferred in younger (<40 years) and older (≥40 years) subjects. The clinical pregnancy rate did not differ between the younger and older subjects (21.6% vs. 23.5%), with similar pregnancy loss rates as well (9.1% vs. 16.7%). These early studies were limited, however, by relatively small sample sizes and the use of procedures less applicable in modern practice such as the transfer of a high number of cleavage stage embryos (mean 4.1 embryos per recipient). In more recent years, an expanding body of literature supports a more important role for endometrial aging in the age-related decline in fertility. A 2012 prospective study by Gupta et al. (Gupta et al., 2012) assessed 270 patients undergoing the transfer of high-quality, untested embryos from egg donors aged 21–31 years. Ongoing pregnancy rates were significantly lower in donor egg recipients aged 40–44 years compared with recipients younger than 40 years (24.6% vs. 38.4%, respectively), and an even greater decrease was observed over the age of 45 years. Although lack of extended culture limited optimal assessment of embryo quality (embryos were transferred at day 2 or 3), these results suggested the possibility of diminished endometrial function with aging.

### 5.1 Pregnancies conceived through assisted reproductive technology (ART)/euploid embryo transfers

Despite continued advancement in ART technologies, including the advent of preimplantation genetic testing for aneuploidy (PGT-A) to select euploid embryos for transfer, live birth rates have not increased substantially in the last decade (Osterman et al., 2023). The relatively low live birth rate of 45%–65% (Cimadomo et al., 2023) following transfer of a euploid embryo indicates that factors other than ploidy are required for successful embryo implantation and pregnancy continuation, including the importance of the endometrium in supporting a healthy implantation.

The “embryo factor” refers to the impact of embryo ploidy on pregnancy rates. Pregnancies conceived from autologous oocytes in women of advanced reproductive age are more likely to be aneuploid (Franasiak et al., 2014) and are at increased risk of miscarriage (Magnus et al., 2019) and pregnancy complications (Pinheiro et al., 2019); it is thus more difficult to separate ovarian and endometrial aging. Though previous studies attempted to minimize the embryo factor by evaluating embryos derived from young oocyte donors, recent studies incorporating extended embryo culture and embryo biopsy for PGT-A followed by transfer of euploid embryos have provided the ability to further reduce the influence of the “embryo factor” and better isolate the endometrial factor. A systematic review and meta-analysis by Vitagliano et al. (Vitagliano et al., 2023) investigated the effect of maternal age on ART success rates after the transfer of euploid embryos derived from autologous oocytes. An analysis of 11,335 ET cycles indicated that the live birth rate (LBR) declined incrementally from the youngest age group (<35 years, LBR 54.8%) to the oldest (>42, LBR 46.2%), further supporting the hypothesis that key changes in endometrial function contribute to decreasing IVF success rates in women aged 35 years or older. Although strengths of this study include the large sample size and the use of narrow age categories, limitations include confounders such as age-related changes in embryos independent of ploidy and the lack of information about uterine pathologies which may be more common with age and which may interfere with embryo implantation.

A systematic review of 18 studies by Zhao et al. investigated the impact of advanced maternal age (AMA), defined as 40 years or older, on endometrial receptivity (Zhao J. et al., 2023). The main objective was to assess the impact of advancing age on implantation, clinical pregnancy, miscarriage and live birth rates in infertile women undergoing ART after oocyte donation. The results showed no significant difference in implantation or clinical pregnancy rates in women with AMA compared to their younger counterparts. A significantly higher miscarriage rate and a lower live birth rate were found in older compared to younger women. Thus, the findings of this study overall suggest a negative impact of age on endometrial function; the results should be interpreted with caution given the heterogeneity in the included studies, the retrospective design of the studies, use of different definitions of AMA, and different endometrium preparation protocols.

### 5.2 Impact of aging on endometrial receptivity and function

Endometrial receptivity refers to the ability of the endometrium to “accept” the embryo for implantation within a specific timeframe of the secretory phase, and to provide an optimal environment for pregnancy development (Bui et al., 2022). A recent systematic review and meta-analysis of studies examining pregnancy outcomes from embryos derived from non-autologous oocytes demonstrated a higher miscarriage rate and lower live birth rate in women with AMA, though the analysis included studies with variable definitions of AMA and a wide range of cutoffs (Zhao J. et al., 2023). A negative correlation has also been noted between advancing age and expression of the implantation marker *HOXA10* in endometrial explants, which is implicated in endometrial-blastocyst communication (Fogle et al., 2010). More recently, Devesa-Peiro et al. (Devesa-Peiro et al., 2022) evaluated endometrial gene expression in women <35 compared to those ≥35 years and identified 5,788 differentially expressed genes between the two groups. Pathway analysis revealed alterations in cilia motility and ciliogenesis pathways, pointing to these cellular changes in the luminal epithelium as some of the crucial dysregulated functions associated with advancing maternal age.

Another factor required for successful embryo implantation is synchronicity of embryonic development relative to the timing of the relatively narrow window of implantation during the secretory phase. Shapiro et al. (Shapiro et al., 2016) investigated whether indicators of endometrial-embryo asynchrony, such as prematurely elevated serum progesterone levels and delayed embryo development were increased in women of advanced reproductive age, defined in this study as 35 years of age and older. Day 5 blastocyst transfer and low serum progesterone levels (i.e., progesterone level lower than 1.5 ng/mL during ovarian stimulation) were defined as markers for a synchronous endometrium. Notably, patients with advanced reproductive age had a significantly lower proportion of synchronous transfers than younger women (31.9% vs. 50.0%). While these results may suggest a more frequently displaced window of implantation in the endometrium of older women undergoing IVF with fresh embryo transfer, this study does not separate the contribution of the embryo (which is more likely to be aneuploid in older subjects) from the contribution of the endometrium even though synchrony is a composite of the two.

### 5.3 Recurrent implantation failure and recurrent pregnancy loss

Clinical entities such as recurrent pregnancy loss (RPL) and recurrent implantation failure (RIF) may be at least partially attributed to an underlying endometrial factor. As such, elucidating the mechanisms behind these pathologies and understanding the role of aging—if any—on their natural history is a topic of interest requiring further investigation. Evaluation of DNA methylation levels, a method known as Horvath’s epigenetic clock, has previously been shown to closely correlate with chronologic age in other human tissues (Horvath, 2013). Olesen et al. (Olesen et al., 2018) used CpG methylation sites to estimate the biological age of the endometrium. Nine patients aged 18–38 years underwent endometrial biopsy in two consecutive menstrual cycles in the mid-secretory phase (7 days after the LH surge). The authors found a significant correlation between chronologic age and biologic age of the endometrium. While this study was limited by a small sample size and lacked pregnancy outcomes data, it provided novel and compelling evidence that the endometrium ages with time.

It has been suggested that a discrepancy between the biological and chronological age of the endometrium (i.e., premature aging of the endometrium) in young women may account for some cases of endometrial factor infertility. A 2022 RNAseq analysis by Chen et al. (Chen et al., 2022) explores this question. In this study, the transcriptomes of 245 women <35 years with history of RIF were evaluated. Twenty-nine women >40 years served as a reference group. All subjects underwent endometrial biopsy in the mid-secretory phase (7 days after the LH surge in a natural menstrual cycle). In the younger cohort, two clusters of subjects were identified based on differential gene expression: cluster 1 was defined by upregulation of pathways involved in immune function while cluster 2 was defined by upregulation of metabolic pathways. The immunologically active cluster had higher expression of genes associated with endometrial receptivity. In contrast, subjects in the metabolically active cluster shared more genes with the reference group (women >40) and had lower implantation rates than those in the immunologically active cluster. Overall, this study suggests that premature aging of the endometrium could contribute to the clinical entity of recurrent implantation failure in younger women with RIF. However, premature aging is a relatively under-studied area of endometrial aging and additional studies are needed to elucidate the causes and characteristics of the prematurely aging endometrium.

### 5.4 Endometrial microbiota, endometrial receptivity

Though long held to be a sterile environment, mounting evidence now supports the existence of a physiologic endometrial microbe population which is impacted by hormonal changes and represents another element of the embryo-endometrial interface (Toson et al., 2022). Multiple studies support a connection between the female reproductive tract microbiome and infertility (Rowe et al., 2020; Cela et al., 2022; Elkafas et al., 2022), but the mechanisms by which changes in the endometrial microbiome contribute to female infertility remain unknown. Further, although the bacterial composition of the endometrium is known to be influenced by age (Fujii and Oguchi, 2023), the role of probiotics and the endometrial microbiota have not been well-studied in the context of age-related decline in fertility.

*Lactobacillus* species are the most common bacteria extending throughout the female reproductive tract, with fewer bacteria in the endometrium compared to the vagina (Chen et al., 2017). A recent study by Fujii et al. (Fujii and Oguchi, 2023) demonstrated changes in the endometrial microbiota with advancing age. In this study, authors analyzed 185 endometrial samples in the mid-secretory phase from infertile patients ranging in age from 25 to 47 years who had a history of at least one prior unsuccessful embryo transfer. Transcriptomic analysis of the endometrium demonstrated that increasing age was associated with a decrease in Lactobacillus-dominant microbiota and the presence of dysbiotic (or disrupted) endometrial microbiota. They also found that patient age and ultra-low biomass were independently associated with risk of pre-receptive endometrium, referring to an endometrium whose transcriptomic profile indicates the window of implantation has not yet been reached, and would thus not be conducive to the implantation of a blastocyst. These findings suggested that the quantity of *Lactobacillus* and its interaction with the host cells in the endometrium play a role in the development of endometrial receptivity. This study did not assess pregnancy outcomes, limiting applicability of the findings.

Other studies have examined the impact of alterations in the vaginal, cervical, and endometrial microbiota on ART outcomes (Cela et al., 2022; Moreno et al., 2022; Fujii and Oguchi, 2023). Women with a lower percentage of vaginal *Lactobacillus* have been found to be less likely to have a successful embryo transfer (Moreno et al., 2022), and a Lactobacillus-dominant microbiota—defined as ≥ 80% of all identified species—is associated with increased implantation, pregnancy, and live birth rates (Moreno et al., 2022). In addition, persistent inflammation of the endometrial mucosa has been shown to impair receptivity through alterations in endometrial decidualization (Di Pietro et al., 2013). In a recent multicenter prospective study (Moreno et al., 2022) a dysregulation in the balance between pathogenic bacteria and non-pathogenic *Lactobacillus* was found to be associated with increased implantation failure. In this study of 342 patients undergoing IVF, the endometrial microbiota was profiled in the endometrial fluid and through endometrial biopsies. *Lactobacillus* was the major genus in both samples and its presence was negatively correlated with the occurrence of pathogenic bacteria. Results showed that patients with a dysbiotic endometrium had increased risk of poor IVF outcomes. Taken together, the findings that increasing age is associated with higher risk of dysbiotic endometrium and that dysbiosis negatively impacts ART outcomes, suggests that adverse effects of an aged endometrium on reproduction may, at least in part, be attributed to microbiome changes. This link, however, remains unproven.

### 5.5 The possible link between oxidative stress and endometrial aging

Oxidative stress (OS) arises from an overabundance of reactive oxygen species (ROS) that overcomes a biological system’s ability to counter them via the production of antioxidant defenses. ROS and antioxidants have been identified in the female reproductive tract, and their populations can be affected by age (Agarwal et al., 2005; Agarwal et al., 2012; Kaltsas et al., 2023). On a molecular level, the negative effects of OS may be the result of lipid damage, inhibition of protein synthesis and depletion of ATP, with reproductive consequences including the destruction of oocytes, altered endocrine function, and endometrial damage, all of which have adverse implications for pregnancy implantation and continuation (Agarwal et al., 2003). If estrogen provides a protective effect against reactive oxygen species (Kaltsas et al., 2023), estrogen may have beneficial effects in terms of antagonizing the impact of oxidative stress. However, this remains theoretical and mechanistic studies are needed. Additional studies exploring the impact of ROS on the aging endometrium are required in order to consider applying estrogen therapy for the treatment of age-related increase in oxidative stress.

### 5.6 Pregnancy complications associated with advanced maternal age

Rodent studies have identified mechanisms by which aging impairs endometrial decidualization. Early studies in the rat compared the decidual response of young and aged ovariectomized rats and found a diminished deciduogenic response in aged rats (Ohta, 1987). Age-related pregnancy complications may originate from endometrial dysfunction leading to abnormal placentation. Indeed, there is an overall increased incidence of spontaneous miscarriage, preeclampsia, gestational diabetes, intrauterine growth restriction, preterm delivery, and stillbirth among women of older reproductive age (Wu et al., 2023). While the exact mechanisms have not yet been elucidated, age-related aberrations in endometrial stromal cell decidualization may play a fundamental role. Decidualization is a requirement for healthy embryo implantation and placentation (Wu et al., 2023). Dysregulated endometrial decidualization is theorized to have the adverse “ripple effect” of recurrent implantation failure and disorders of pregnancy, including preeclampsia and recurrent miscarriages (Agarwal et al., 2012; Garrido-Gomez et al., 2017; Wu et al., 2023). Many of these complications may be considered problems of endometrial aging as abnormal decidualization, placentation and subsequent pregnancy complications exist on a continuum (Conrad, 2020). Thus, further elucidating mechanisms underlying the adverse effects of age on stromal cell decidualization and endometrial remodeling may impact prevention of age-related complications of pregnancy (Wu et al., 2023).

## 6 Discussion

Female reproductive aging, and attempts to circumvent the process, have been a major focus of reproductive research for decades. While it has been well established that ovarian aging is the most significant contributor to the age-related decline in female fertility, the endometrium has been comparatively overlooked. Although the endometrium is a self-regenerating tissue, evidence points to senescence over time in the individual cellular compartments. Additionally, as it is a hormone-responsive tissue, it is susceptible to the changing hormonal milieu that is part and parcel of reproductive aging. As highlighted in this review, the individual cell types in the endometrium (stromal, epithelial, vascular, immune and stem) are subject to the forces of aging and senescence. However, there is a paucity of studies to determine mechanisms of endometrial aging and its clinical sequelae; many of the studies are retrospective in nature, report small sample sizes, have not been validated in other studies, or have not been replicated in humans. Furthermore, the clinical and translational studies examined in this review employ variable definitions of advanced age—ranging from 35 to >40 years of age—while other explore changes related to the menopausal transition which typically occurs at age 50. In light of this, firm criteria cannot be established to define advanced age with respect to the endometrium. However, collectively, the studies reviewed herein highlight age-related molecular and cellular changes that support the concept that endometrial aging may play a significant role in overall reproductive aging.

There exist several possible avenues for future investigation. Beginning with the ovary, in addition to steroid hormones such as estradiol and progesterone, the ovary is a rich source of peptide hormones inhibin A, inhibin B, activin and others, which regulate the pituitary-ovarian feedback loop (Muttukrishna et al., 2000). While ovarian aging has been examined mostly in terms of the diminishing pool of available oocytes, the reduced follicular mass also produces fewer peptide hormones which may have direct implications for the endometrium. Activin receptors have been described in the endometrium (Mabuchi et al., 2010), and all of these ovarian peptide hormones have been found to decrease with age (Muttukrishna et al., 2000). Following ovulation, the corpus luteum secretes estradiol, progesterone and inhibin A, and the age-related decline in luteal function may also affect the quality of the secretory endometrium (Broekmans et al., 2009). Although this does not represent an active area of research, these studies highlight a gap in the current literature and an opportunity to examine the effects of the aging ovaries on the endometrium more closely. Finally, studies in other tissues or organ systems may ultimately help to inform our understanding of endometrial cellular compartments and their aging process. For example, although age-related changes in endometrial vasculature have not been well studied, evidence from other regenerative tissues provides insight into the process of endothelial cell aging that may inform our understanding of endometrial vascular aging. In the gastrointestinal tract, for example, rodent studies demonstrate an age-associated reduction in blood flow to the gastric mucosa (Tarnawski et al., 2007) and endothelial cells obtained from the gastrointestinal tract of aging rats demonstrate decreased angiogenesis (Ahluwalia et al., 2014). Studies such as these may suggest productive areas of investigation with respect to endometrial aging.

Aging of endometrial stem cells, and/or harnessing endometrial stem cells as potential therapeutics for the aging endometrium, are other potential areas of research. Although studies are currently investigating the use of stem cells to promote endometrial regeneration in women with intrauterine adhesions or tissue injury (Gargett et al., 2012; Strug and Aghajanova, 2021), the lessons about endometrial damage, repair and regeneration may have applications in the study of endometrial aging. Various sources of stem cells exist for this purpose. Menstrual blood stem/progenitor cells can be collected in menstrual cups and cultured, representing an easily accessible source of stem cells (Bozorgmehr et al., 2020). The findings of age-related changes in endometrial stem cells, and the possibility that these same cells can be harnessed to repair age-related change, should lead to many fruitful opportunities for investigation. In addition to exploring the diagnostic and therapeutic potential of stem cells, elucidating the requirements for endometrial receptivity may yield answers relevant to aging. In addition, the concept of endometrial “sensitivity”, which was historically proposed to describe the capacity of the endometrium to respond to steroid hormones, prostaglandins, and vasoactive mediators (Psychoyos, 1984), may be susceptible to age-related changes. The impact of endometrial aging on sensitivity and receptivity, however, is as yet unclear.

Finally, several new and emerging interventions may have the potential to contribute to the study and treatment of endometrial aging. As prior studies have suggested a link between thin endometrium and premature epithelial cell senescence (Lv et al., 2022), treatment options that currently target thin endometria may in the future be applied to mitigating the effects of endometrial aging. Platelet-rich plasma has been investigated as a treatment for thin endometrium and appears to be effective in subjects of older reproductive age (Gangaraju et al., 2023) though studies such as this one have very small sample sizes (fewer than 10 subjects) and firm conclusions cannot be drawn.

The studies reviewed herein present the scope of the endometrial aging question, from endometrial physiology and regeneration to an exploration of the age-related changes occurring in the individual cell types, concluding with the implications of endometrial aging that can be inferred from clinical studies. While early studies in this field examined the process of aging through an ovary-centric lens, this review highlights the evidence that the biological and chronological age of the endometrium may play a role in pregnancy success. This review highlights important mechanisms that may underlie the adverse impact of an aging endometrium on fertility; however, we do not as yet have definitive molecular or cellular markers to define aged endometrium. Future studies should continue to explore these important facets of the reproductive aging paradigm in order to better understand their origins and more effectively develop therapeutic options.
